# Rural-urban differentials in pregnancy-related mortality in Zambia: estimates using data collected in a census

**DOI:** 10.1186/s12963-015-0066-9

**Published:** 2015-11-30

**Authors:** Richard Banda, Knut Fylkesnes, Ingvild Fossgard Sandøy

**Affiliations:** Central Statistical Office, Lusaka, Zambia; Centre for International Health, University of Bergen, Bergen, Norway

**Keywords:** Maternal mortality, Pregnancy-related mortality, Census, Rural-urban, Zambia

## Abstract

**Background:**

The use of census data to measure maternal mortality is a recent phenomenon, implemented in settings with non-functional vital registration systems and driven by needs for trend data. The 2010 round of population and housing censuses recorded a significant increase in the number of countries collecting maternal mortality data. The objective of this study was to estimate rural-urban differentials in pregnancy-related mortality in Zambia using census data.

**Methods:**

We used data from the Zambia 2000 and 2010 censuses. Both censuses recorded the female population by age, the number of children ever born, and live births 12 months prior to the census. The 2010 census further recorded, by age, household, and pregnancy-related deaths 12 months prior to the census. We evaluated and adjusted recorded live births using the cohort Parity Fertility ratio method, and household deaths using deaths distribution methods (General Growth Balance and Synthetic Extinct Generation). Adult female mortality and pregnancy-related mortality for rural and urban areas were estimated for the period October 2009 to October 2010.

**Results:**

Data evaluation showed errors in recorded population age, age-at-death, live births, and deaths, and appropriate adjustments were made. Adjusted adult female mortality was high; an adolescent aged 15 years had a one-in-three chance of dying before her 50th birthday in rural areas and one-in-four chance in urban areas. Pregnancy-related deaths comprised 15.3 % of all deaths among reproductive-age women overall; 17.9 % in rural areas and 9.8 % in urban areas. The pregnancy-related mortality ratio for the period was 789 deaths/100,000 live births overall: 960/100,000 live births in rural areas and 470/100,000 live births in urban areas.

**Conclusions:**

Census-based estimates show very high adult female mortality and particularly high pregnancy-related mortality in both rural and urban areas of Zambia 12 months prior to the 2010 census. Future censuses should pay greater attention to strategies for improving data quality.

## Background

The millennium development goals (MDGs) related to health have pushed systems of data collection in many low- and middle-income countries into overdrive as global and national policymakers demand regular updates on progress towards achieving them. The fifth MDG focuses on maternal health, and one of the targets is to reduce maternal mortality ratio (MMRatio) by three-quarters between 1990 and 2015 [1]. Continuous civil registration systems provide an ideal source of information on deaths and cause of death, including maternal mortality [2–4]. However, less than half of countries globally have complete civil registration systems with adequate cause of death information [4]. As a result, many countries rely on population-based surveys to monitor MDG indicators. Unless verbal autopsy is included to enable ascertainment of actual maternal deaths, these surveys only provide measures of pregnancy-related mortality. The use of a census to measure pregnancy-related mortality is a recent phenomenon, resulting from the need to establish long-term trend data, as well as estimates with much more recent reference periods. An increase in the number of countries including questions on pregnancy-related mortality has been noted following the United Nations (UN) recommendation for this to be a core topic in the 2010 round of censuses [5].

Due to the lack of a functional civil registration system, Zambia has relied on survey data generated using the sisterhood method in the Zambia Demographic and Health Surveys (ZDHS). Recent survey estimates of the pregnancy-related mortality ratio indicate declining trends; 729 (95 % CI: 586–872) in the 2001/2 survey, 591 (95 % CI: 450–732) in the 2007 survey and 389 (95 % CI: 323–474) in the 2013/14 survey [6]. These estimates refer to a period 0–7 years prior to each survey and are only available at national level due to sample size limitations.

The advantage of the census approach lies in the ability to decompose estimates to subnational levels such as rural and urban areas [4, 7]. Subnational estimates are important in understanding levels of maternal mortality and its drivers within individual countries. The national requirement for subnational estimates was one of the key factors leading to the inclusion of pregnancy-related mortality in the Zambia 2010 census. The scope and primary focus of a census have given rise to concerns on whether it can succeed as a source of quality pregnancy-related mortality data [8]. The strengths and weaknesses of census-based approaches for estimating pregnancy-related mortality have been highlighted in several studies [7–14]. When household deaths also are included in the census, the level of mortality during the reproductive age period can be used as a plausibility check on the estimated level of pregnancy-related mortality. The main objective of this study was to estimate pregnancy- related mortality differentials between rural and urban areas in Zambia using census data and to capture this against prevailing adult female mortality differentials.

## Methods data sources

We used data collected in the Zambia 2000 and 2010 censuses of population and housing. The two censuses were conducted exactly 10 years apart, each with October16th as the census reference night. Each census was preceded by an extensive cartographic mapping exercise aimed at subdividing the country into standard enumeration areas (SEAs) with between 80 and 150 households. An enumerator was assigned to each SEA for the purpose of census enumeration. For the 2010 census, about 25,000 enumerators took part in the data collection exercise [15]. The census estimated a total population (de jure) of 13,092,666, of which 39.5 % resided in urban areas [15]. Females made up 50.7 % of the total population, and 47.2 % of the female population was in the reproductive age group 15–49 [15].

## Data evaluation

### Age

Age data in the two censuses was collected using the question on age at last birthday. Errors in age data are common in censuses, but tend to be more problematic in populations where literacy levels are low [16]. Dynamic “hot deck” imputation was used to generate values for missing ages during data processing of both the 2000 and 2010 censuses. We still evaluated the quality of age data using the Age-sex accuracy index (ASAI) [16]. The ASAI is a summary of the age and sex ratio scores computed using data for 5-year age groups from 10–14 through 65–69. The index only provides a measure of the quality of age data as follows: An index of <20 means age-sex data is accurate; an index of 20–40 means age-sex data is inaccurate, and an index of >40 means age-sex data is highly inaccurate [16]. To compute the ASAI, we used the spreadsheets AGEMSTH developed by the United States Census Bureau [17].

### Live Births and children ever born

Data on children ever born were collected from all women aged 12 years and older, while data on live births in the 12 months prior to the census were collected from women aged 12–49 [18, 19]. Such data are often affected by reporting errors due to omission of children who die in infancy or who live elsewhere at the time of census [16, 20]. Detailed questions were used, including asking about children that had since died or lived elsewhere, in order to reduce such errors [18, 19]. We applied the 10-year inter-survey synthetic cohort PF Ratio method to assess the quality of data and generate adjustment factors for live births [20].

### Household deaths

Zambia collected data on household deaths in the census for the third time during the 2010 census, after two unsuccessful attempts in the 1969 and 1990 censuses [21, 22]. Households reported on deaths of household members 12 months prior to the census. Information on age at death, sex of the deceased and cause of death were collected for all reported deaths. Misreporting (omissions and duplications) is common for such data, including erroneous recording of age at death and sex. Similar to population age, missing values for age at death were generated using dynamic hot deck imputations during data processing. We evaluated the quality of age at death data using ASAI. We further evaluated the completeness of recorded deaths using deaths distribution methods (DDM), the General Growth Balance (GGB) [23, 24], the Synthetic Extinct Generation (SEG), and the combined GGB-SEG [23]. Both the SEG and GGB require a population closed to migration (or with negligible migration) and accurate recording of age for both population and deaths [23]. The SEG further requires that population coverage is constant across age and in each of the two censuses [23]. We first used the GGB to estimate coverage of deaths and population. We then applied the SEG to estimate coverage of deaths, and checked the estimates with those obtained from the GGB. As part of the SEG procedure, life table deaths were estimated using observed deaths and growth rates at each age. We finally applied the combined GGB-SEG in order to adjust for population coverage between the two censuses. Final estimates of mortality were adjusted based on the combined GGB-SEG using age-trims 5+ to 65+. Given the high level of mortality in early adulthood in Zambia, we opted against fitting the age-trims 30+ to 65+ as that would exclude a huge number of female deaths before age 30. Hill and colleagues recommended the use of the combined GGB-SEG approach fitted to the age-trims 5+ to 65+ for optimal results of the conditional probability of dying between exact age 15 and 60 (_45_*q*_15_) [25]. To apply the data evaluation methods, we used the spreadsheets developed by the World Health Organization (WHO) for the estimation of pregnancy-related mortality in a census [23].

### Pregnancy-related deaths

The WHO defines a maternal death as one that occurs while a woman is pregnant, during childbirth, or within 42 days of termination of pregnancy from a cause directly related to or aggravated by the pregnancy or its management and not an accidental or incidental cause [26]. In the 2010 census, all deaths of females aged 12–49 years reported to have taken place in the 12 months prior to the census attracted further probing to determine the time of death relative to the pregnancy state. The first probing question was: “Did the death occur while pregnant?” If the answer was “no” to this question, the respondent was asked: “Did the death occur during childbirth?”, and if the answer was “no” to this too, a third question was posed: “Did the death occur during the 6-week period following the end of pregnancy, irrespective of the way the pregnancy ended?” [15]. A “yes” to any of the three questions was used to estimate the number of pregnancy-related deaths. No attempt was made to ascertain whether the cause of death was actually related to the pregnancy, and thus “pregnancy-related deaths” is a more appropriate term than maternal deaths.

Formal methods for assessing the accuracy of pregnancy-related deaths (PR deaths) recorded in a census are unavailable [23]. We used proxy methods to evaluate the plausibility of pregnancy-related deaths recorded:

i. We first reviewed the numbers of deaths recorded overall and within each 5-year age group.
ii. We computed and reviewed the proportions of pregnancy-related deaths based on reported time of death.
iii. We computed and reviewed the proportions of total female deaths that were pregnancy-related (PMDF) overall and within each 5-year age group.
iv. We computed and reviewed the percent share of pregnancy-related deaths within each 5-year age group.
v. We finally computed and reviewed the crude pregnancy-related mortality ratio (PRMRatio) overall and for each of the 5-year age group.

We applied three options in estimating the PRMRatios; no adjustment, partial adjustment (deaths only), and full adjustment (both deaths and live births). In the first option, deaths and live births as recorded in the 2010 census were used to estimate the PRMRatios. In the second option, adjustment was made to deaths using adjustment factors from the combined GGB- SEG. Pregnancy-related deaths were adjusted on the assumption that coverage was similar to that estimated for all female deaths by the combined GGB-SEG within rural or urban areas. In the third option, adjustment was further made to recorded live births using the average of PF ratios for age groups 20–24, 25–29 and 30–34. The average of the three age groups was considered a robust indicator of the level of completeness of births reporting in the census and was arrived at after analyzing the distribution of the PF ratios by age group.

### Ethical approval

Both censuses were conducted under the Census and Statistics Act 127 of the laws of Zambia [27]. The Central Statistical Office is mandated to conduct censuses and surveys as prescribed by the Act and guided by national and international requirements for data on Zambia. The data used in this study are publicly available in a series of census tabulation reports [28]. Use of such data does not require ethical approval [29].

## Results

The evaluation results indicated errors in the data on age; ASAI values of 34.2 and 38.5 were estimated for population age distributions recorded in rural and urban areas, respectively during the 2000 census. Marginal improvements in age reporting were registered in the 2010 census; ASAI values of 31.2 and 36.6 were estimated for the population age distributions in rural and urban areas respectively. Therefore age data from both censuses could be classified as inaccurate. Further evidence of this was found in age heaping, common in both censuses at ages ending in 0, 2, 5, and 8. The ASAI values for age at death were much higher, 48.7 for rural areas and 65.7 for urban areas respectively, indicating highly inaccurate age-at-death data. We adjusted both the age distributions of the population and deaths by smoothing with the Arriaga technique, a method that applies mild smoothing and maintains the original distribution totals [17].

The numbers of reproductive age women, children ever born, and live births 12 months prior to the census are presented in Table 1, together with the results of the cohort PF ratio method. Results indicated higher cohort parities (completed fertility) compared to cumulated “current” fertility at each age. The age-specific PF ratios were greater than unity in both rural and urban areas, indicating underreporting of live births and a need for upward adjustment. The adjusted number of live births 12 months prior to the 2010 census was 549,000 total; 357,784 in rural areas and 191,216 in urban areas. For the same period, the observed and adjusted total fertility rates (TFR) were 5.67 and 6.90 for rural areas, and 3.37 and 4.36 for urban areas respectively.

**Table 1:** Results of the application of the 10-year inter-survey synthetic cohort PF ratio method to fertility data for rural and urban areas for the intercensal period 2000–2010

	Rural															
	2000 census					2010 census					Application of cohort PF ratio method					
Age Group	Number of Women	Children Ever Born Alive	Children Born in last year	Age-Specific Fertility Rates	Average Parity P	Number of Women	Children Ever Born Alive	Children Born in last Year	Age-Specific Fertility Rates	Average Parity P	Parity Change	Synthetic Cohort Parity	Age-Specific Fertility Rates	Cumulated Fertility to Age x	Parity Equivalent F	Ratio P/F
15–19	328,766	113,801	37,364	0.114	0.35	418,087	121,237	41,899	0.100	0.29	0.290	0.29	0.107	0.00	0.26	1.10
20–24	283,827	456,590	72,720	0.256	1.61	339,832	539,787	84,736	0.249	1.59	1.588	1.59	0.253	0.53	1.29	1.23
25–29	232,022	685,824	56,057	0.242	2.96	277,415	897,227	72,639	0.262	3.23	2.888	3.18	0.252	1.80	2.57	1.24
30–34	171,981	749,596	36,379	0.212	4.36	219,798	970,606	47,489	0.216	4.42	2.807	4.40	0.214	3.06	3.71	1.18
35–39	134,471	778,153	23,385	0.174	5.79	174,947	977,140	31,641	0.181	5.59	2.630	5.81	0.177	4.13	4.69	1.24
40–44	102,396	697,947	9268	0.091	6.82	132,609	790,430	12,286	0.093	5.96	1.602	6.00	0.092	5.01	5.31	1.13
45–49	83,693	570,474	2882	0.034	6.82	104,276	695,798	3441	0.033	6.67	0.886	6.69	0.034	5.47	5.42	1.24
				(Observed TRF)					(Observed TFR)				(Intercensal TFR)			
Total	1,337,156	4,052,385	238,055	5.61		1,666,964	4,992,225	294,131	5.67				5.64			
Urban																
15–19	210,914	46,597	14,270	0.068	0.22	323,150	54,574	17,100	0.053	0.17	0.169	0.17	0.060	0.00	0.14	1.18
20–24	195,623	224,804	31,597	0.162	1.15	291,998	282,823	43,534	0.149	0.97	0.969	0.97	0.155	0.30	0.77	1.27
25–29	160,364	347,923	24,210	0.151	2.17	245,106	522,434	42,062	0.172	2.13	1.911	2.08	0.161	1.08	1.57	1.32
30–34	108,011	382,196	14,464	0.134	3.54	184,220	582,924	26,577	0.144	3.16	2.015	2.98	0.139	1.88	2.32	1.29
35–39	79,602	397,839	7671	0.096	5.00	138,963	548,664	13,811	0.099	3.95	1.779	3.86	0.098	2.58	2.90	1.33
40–44	58,991	362,437	2414	0.041	6.14	91,831	420,391	3864	0.042	4.58	1.039	4.02	0.041	3.07	3.20	1.26
45–49	42,351	269,331	678	0.016	6.36	66,771	384,966	998	0.015	5.77	0.768	4.63	0.015	3.28	3.25	1.42
				(Observed TRF)					(Observed TFR)				(Intercensal TFR)			
Total	855,856	2,031,127	95,304	3.34		1,342,039	2,796,776	147,946	3.37				3.35			

**Fig. 1:**
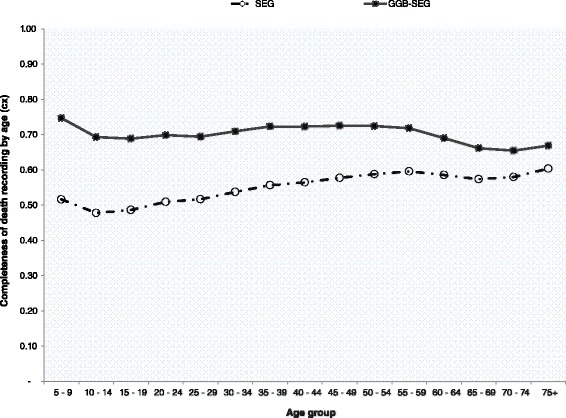
Completeness of deaths recording by age: results of the SEG & combined GGB- SEG for rural Zambia

**Fig. 2:**
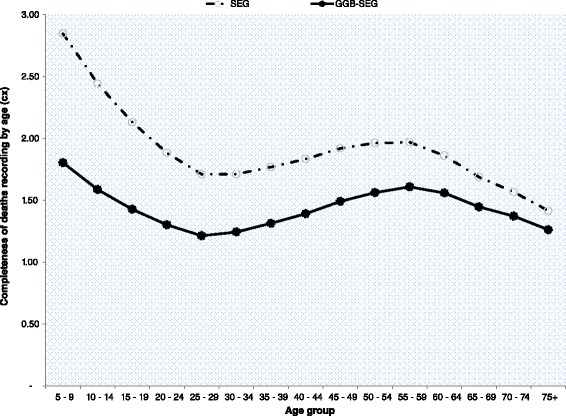
Completeness of deaths recording by age: results of the SEG & combined GGB- SEG for urban Zambia

A total of 76,692 female deaths (of which 26,427 deaths were of women aged 15–49) were recorded for the 12 months prior to the 2010 census. In rural areas 47,554 females deaths were recorded and 13,640 deaths were of women aged 15–49, while in urban areas 29,138 female deaths in total and 12,786 deaths of women aged 15–49. For rural areas, all three methods of evaluation (GGB, SEG, and combined GGB-SEG) indicated underreporting of female deaths, while in urban areas the three methods indicated over-reporting of deaths (Tables 2 and 3). In rural areas, the percentage point difference between the GGB and SEG estimates of deaths coverage was 18 %, twice the difference in urban areas. For both rural and urban areas, deaths coverage was differential by age as observed from the plots of the SEG and combined GGB-SEG (Figs. 1 and 2) showing that the estimates were not aligned along a straight line. (The same was also the case for the GGB plots; data not shown.) However, deaths coverage by age was more stable in rural areas compared to urban areas (where the line was highly curvilinear; see Fig. 2). The coverage estimates from the combined GGB-SEG were used to adjust recorded deaths. Summary measures of adult mortality are also provided in Tables 2 and 3. The estimated number of life table deaths in early adulthood (before age 40) was higher than in late adulthood (age group 40–60) in both rural and urban areas. However, the probability of dying was high in late adulthood relative to the probability of dying in early adulthood. The probability of dying between ages 15 and 50 was 39 % in rural areas and 25 % in urban areas, while the probability of dying between ages 15 and 60 was 50 % in rural and 34 % in urban areas.

**Table 2:** Summary results of data evaluation and estimated adult female mortality for the period October 2009 to October 2010, Zambia

	Summary result of mortality data evaluation						
Statistic	Age range	Rural			Urban		
		GGB	SEG	GGB-SEG	GGB	SEG	GGB-SEG
Slope		1.382			0.56		
Intersection		0.008			−0.007		
K1:k2	5+ to 65+	1.078			0.934		
Coverage		0.724	0.548	0.712	1.785	1.876	1.394

**Table 3:** Summary results of data evaluation and estimated adult female mortality for the period October 2009 to October 2010, Zambia

	Summary measures of adult female mortality							
Indicator	Rural				Urban			
_35_q_15_	Crude	GGB	SEG	GGB-SEG	Crude	GGB	SEG	GGB-SEG
	0.287	0.373	0.460	0.389	0.337	0.205	0.196	0.248
_45_q_15_	0.375	0.477	0.576	0.496	0.449	0.284	0.272	0.339
_20_q_20_ /_20_q_40_	0.833	0.839	0.847	0.841	0.711	0.694	0.693	0.699
_30_q_10_ /_20_q_40_			1.63	1.38			0.98	1.13
_20_q_20_ /_20_q_40_			1.32	1.15			0.88	1.00

**Fig. 3:**
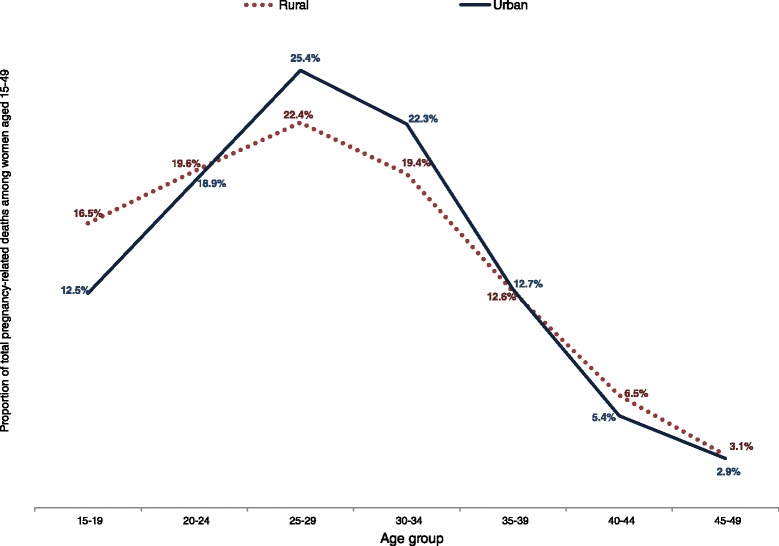
Percent of total pregnancy-related deaths for each 5-year age group within rural and urban areas

**Fig. 4:**
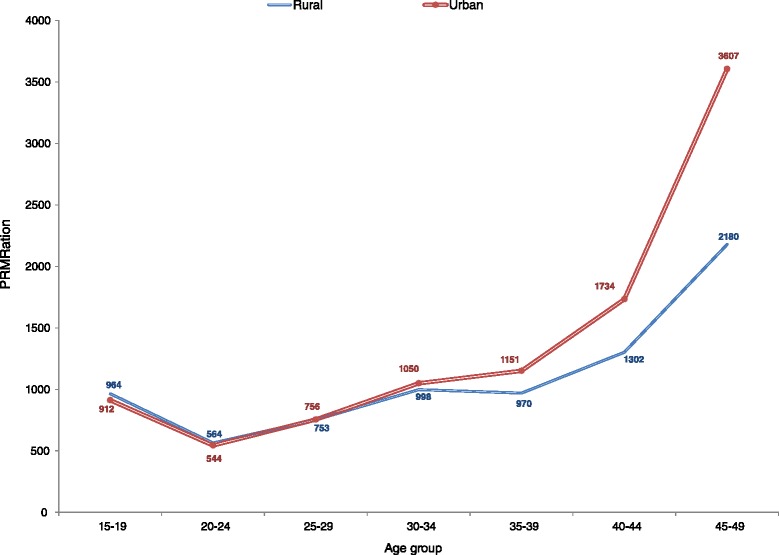
Age-specific crude PRMRatios by rural and urban residence

About half of all recorded pregnancy-related deaths were reported to have occurred in the antepartum period (while the woman was pregnant); 48 % in rural areas and 50 % in urban areas, while postpartum deaths constituted 28 % in rural areas and 17 % in urban areas. A total of 2445 pregnancy-related deaths of women aged 15–49 were recorded in rural areas, representing a PMDF of 17.9 %, while in urban areas, 1252 pregnancy-related deaths were recorded, representing a PMDF of 9.8 % (Table 4). Pregnancy-related deaths as a proportion of total deaths of women was highest among young women aged 15–19, particularly in rural areas where the PMDF in this age group was 44 % compared to 17 % in urban areas. However, women in the age group 25–29 had the highest proportion of total pregnancy-related deaths recorded in both rural and urban areas (Fig. 3). The crude PRMRatio was marginally higher in urban areas; 846/100,000 live births compared to 831/100,000 live births in rural areas (Table 4). The age-specific crude PRMRatios were higher among urban women aged 15–19 and older than 35 years compared to their rural peers (Fig. 4). Adjustment of both deaths and live births (full adjustment) resulted in a PRMRatio of 789/100,000 live births overall; 960/100,000 live births in rural areas and 470/100,000 in urban areas.

**Table 4:** Pregnancy-related mortality using different adjustment options; option I (No adjustment), option II (Partial adjustment-deaths only) and option III (Full adjustment-both deaths and births)

Option I (No adjustment)						Option II (Partial adjustment)					Option III (Full adjustment)				
Zambia total															
Age group	Recorded Deaths	Recorded PR Deaths	Recorded Live Births	Crude PRMRatio	Crude PMDF	Adj. Deaths	Adj. PR Deaths	Recorded Live Births	Partially Adj. PRMRatio	Adj. PMDF	Adj. Deaths	Adj. PR Deaths	Adj. Live Births	Adj. PRMRatio	Adj. PMDF
15–19	1834	560	58,999	949	0.31	1953	680	58,999	1152	0.35	1953	680	73,068	930	0.35
20–24	4611	715	128,270	557	0.16	4981	842	128,270	656	0.17	4981	842	159,340	528	0.17
25–29	4969	865	114,701	754	0.17	5320	997	114,701	869	0.19	5320	997	142,723	698	0.19
30–34	5144	753	74,066	1017	0.15	5481	866	74,066	1170	0.16	5481	866	92,116	940	0.16
35–39	4449	466	45,452	1025	0.10	4747	546	45,452	1200	0.11	4747	546	56,339	968	0.11
40–44	3038	227	16,150	1406	0.07	3277	273	16,150	1690	0.08	3277	273	19,939	1369	0.08
45–49	2382	111	4439	2501	0.05	2582	131	4439	2956	0.05	2582	131	5476	2397	0.05
Total	26,427	3697	442,077	836	14.0 %	28,342	4334	442,077	980	15.3 %	28,342	4334	549,000	789	15.3 %
Zambia Rural (Adj. factors: deaths = 1.41; births = 1.22)															
15–19	927	404	41,899	964	0.44	1303	568	41,899	1355	0.44	1303	568	50,966	1114	0.44
20–24	2431	478	84,736	564	0.20	3417	672	84,736	793	0.20	3417	672	103,074	652	0.20
25–29	2553	547	72,639	753	0.21	3588	769	72,639	1058	0.21	3588	769	88,359	870	0.21
30–34	2603	474	47,489	998	0.18	3658	666	47,489	1403	0.18	3658	666	57,766	1153	0.18
35–39	2260	307	31,641	970	0.14	3176	431	31,641	1364	0.14	3176	431	38,488	1121	0.14
40–44	1596	160	12,286	1302	0.10	2243	225	12,286	1830	0.10	2243	225	14,945	1505	0.10
45–49	1270	75	3441	2180	0.06	1785	105	3441	3063	0.06	1785	105	4186	2518	0.06
Total	13,640	2445	294,131	831	17.9 %	19,171	3436	294,131	1168	17.9 %	19,171	3436	357,784	960	17.9 %
Zambia Urban (Adj. factors: deaths = 0.72; births = 1.29)															
15–19	906	156	17,100	912	0.17	650	112	17,100	654	0.17	650	112	22,101	506	0.17
20–24	2180	237	43,534	544	0.11	1564	170	43,534	390	0.11	1564	170	56,266	302	0.11
25–29	2416	318	42,062	756	0.13	1733	228	42,062	542	0.13	1733	228	54,364	420	0.13
30–34	2541	279	26,577	1050	0.11	1822	200	26,577	753	0.11	1822	200	34,350	583	0.11
35–39	2189	159	13,811	1151	0.07	1570	114	13,811	826	0.07	1570	114	17,850	639	0.07
40–44	1442	67	3864	1734	0.05	1034	48	3864	1244	0.05	1034	48	4994	962	0.05
45–49	1112	36	998	3607	0.03	798	26	998	2587	0.03	798	26	1290	2002	0.03
Total	12,786	1252	147,946	846	9.8 %	9171	898	147,946	607	9.8 %	9171	898	191,216	470	9.8 %

## Discussion

Census-based estimates show very high adult female mortality and particularly high pregnancy-related mortality in both rural and urban areas of Zambia. Adjusted mortality was particularly high in rural areas. A woman aged 15 in rural areas had a chance of dying before her 50th birthday of one-in-three compared to one-in-four for her peer in urban areas. The chance of dying before her 60th birthday increased to *one-in-two* in rural areas and one-in- three in urban areas. In rural areas, one-in-six deaths of women aged 15–49 was pregnancy- related and in urban areas, the proportion was one-in-ten. The adjusted PRMRatios were 960/100,000 live births and 470/100,000 live births in rural and urban areas respectively.

Direct measurement of adult mortality in Zambia using household deaths collected in a census has not been successful prior to the 2010 census due to poor field implementation and questionnaire designs. In the 1969 census, enumerators confused questions on household deaths with questions on dead children among those ever born to reproductive-age women [21]. In the 1990 census, a question on household deaths 12 months prior to the census, disaggregated by sex, was added to the questionnaire, but a question on age at death was missing [22]. The 2010 census was the first attempt to measure pregnancy-related mortality in the census [15]. This study shows both the feasibility and challenges of measuring mortality in a census. The census succeeded in providing estimates of adult female mortality overall, and pregnancy-related mortality. For both rural and urban areas, adult female mortality overall and pregnancy-related mortality were high, before and after adjustment for coverage errors. When compared with other estimates for Zambia from the same time period, the census estimates are much higher. For example, the census PMDF of 15.3 % is much higher than the UN estimate of 9.1 % for the year 2010, and the census PRMRatio is high compared to the UN estimated MMRatio of 440/100,000 live births (95 % CI: 220–790) [30]. These differences partly arise from the UN estimates being based on modeled data from a number of sources, and from the fact that the UN modelling includes downward adjustment of reported pregnancy-related deaths to arrive at an estimate of actual maternal deaths. The census estimates are also higher than the estimated PMDF of 9.5 % from the 2013/14 ZDHS for the period 2007–2013 [6]. This discrepancy was somewhat surprising because a comparison by Hill and colleagues of the household deaths and sibling history approaches to measuring pregnancy-related mortality applied to surveys found close agreement in the estimates of the PRMRatios [31]. When age distributions of the PMDF are compared, possible sources for the difference between the census and DHS overall estimate of PMDF emerge. The census PMDF was 35 % for women aged 15–19 and 19 % for women aged 25–29 compared with very low estimates of 4 and 7 % respectively from the 2013/14 ZDHS. Since data on pregnancy-related mortality both from the census and the DHS have substantial limitations and neither data source can be regarded as a gold standard, we are unable conclude which of the estimates are most likely to be valid.

One potential reporting problem affecting both a census and surveys pertains to misclassification of deaths. Maternal deaths are known to be concentrated around childbirth or soon after and the majority are usually due to hemorrhage [32–34]. In the 2010 census the majority of pregnancy-related deaths were, however, concentrated in the antepartum period, in both rural and urban areas. It is possible that the sequenced order of asking about the timing of death could have biased the final tally. The use of three questions in a sequence was meant to improve recall and hence improve the quality of data collected. However, no formal validation has been done to determine how well the questions worked, including assessing whether the order of the questions affects the level of reporting bias [35]. A study in Bangladesh found misclassification of the timing of deaths when responses given to direct household questions were checked against information collected using verbal autopsy; 20 % of pregnancy-related deaths were found to have been misclassified by households as having occurred during pregnancy as opposed to having occurred during delivery [36]. This indicates the need to further validate these questions before use in future censuses to reduce misclassifications of deaths.

The adjusted PRMRatios gave more plausible rural-urban differentials than the crude estimates given the challenging reproductive health situation in rural areas. Early childbearing is more common in rural areas, where 36 % of young women aged 15–19 have initiated childbearing compared to 20 % in urban areas [6]. Pregnant adolescents have poor access and utilization of antenatal care services [37], increasing the risk of maternal death if they develop complications. Only 56 % of women deliver in health facilities in rural areas and 52 % have skilled attendants during delivery compared to 89 % of women delivering in health facilities, and 88 % having the assistance of skilled attendants, in urban areas [6]. Long distances to health facilities and lack of health workers prevent women in rural areas from accessing maternal health services [38–42]. Endemic malaria, anemia, and malnutrition, which are more prevalent in rural areas, also contribute to the high pregnancy-related mortality in Zambia.

In a high-prevalence country like Zambia, HIV/AIDS has been found to be the major cause of young adult female mortality and thus likely to substantially affect pregnancy-related mortality [43]. The HIV prevalence was 21 % in urban areas and 10 % in rural areas of Zambia in 2013–14 among women aged 15–49 [6], and the UN estimates between 15 and 30 % of maternal deaths in Zambia to be a result of HIV infection [4, 30]. Due to elevated mortality, mainly from the HIV/AIDS epidemic, the UN projected life expectancy at birth of 45.6 years for the period 2010–2015 is 10 years lower than it would be in the absence of HIV/AIDS [44]. The census estimates of the adult conditional probabilities of dying before age 50 (_35_q_15_) and before age 60 (_45_q_15_) were somewhat high, but plausible in depicting the prevailing level of mortality due to HIV/AIDS, and the observed high mortality in early adulthood relative to mortality in late adulthood could be a reflection of the impact of the epidemic [43, 45, 46]. The scaling up of free anti-retroviral therapy in public health facilities started around 2004–05 and is likely to have reduced mortality somewhat. However, by 2010, coverage was well below 50 % and with still substantial HIV-related mortality [47].

Evaluation studies of census-based measurement of pregnancy-related mortality emphasize the need for rigorous data evaluation before making any estimates [8]. We applied standard data evaluation methods for use with census data. Results indicated errors in recorded population age, age at death, live births, and deaths; hence adjustments to correct for the observed deficiencies in the data were necessary before final estimates could be generated. Where data quality is high, adjustments can be avoided as they could introduce their own biases [16]. Both the GGB and GGB-SEG showed too high coverage of urban female deaths; 1.79 and 1.39 respectively. However, coverage of rural female deaths was low; 0.72 and 0.71 for the GGB and GGB-SEG, respectively. Although under-reporting of deaths is usually more likely, over-reporting is equally possible. Hill and colleagues found as much as 20–30 % over-reporting of deaths in the Nicaragua and Paraguay censuses using the GGB [11]. Over-reporting was attributed to possible confusion with the reference periods [11].

Confusion with the reference period is also possible in the Zambia 2010 census since deaths were recorded for the period October 2009 to October 2010, and not a proper calendar year.

However, this would have affected all household deaths and not only urban female deaths. Over-reporting of deaths can result from the way funerals are conducted in Zambia, especially in urban areas. A death usually attracts funeral gatherings for days and it is not uncommon for such gatherings to be hosted away from the usual place of residence of the deceased person.

Funeral hosting may be determined by several factors, including the capacity to meet funeral expenses. In such a case, the census might record the same death twice at two different homes. If there was no systematic over-reporting of urban female deaths in the 2010 census, coverage would be similar for male and female deaths. The GGB estimate for urban males was 0.97 and the GGB-SEG was 0.86, which indicates lower coverage of male than female deaths. It was possible for the high coverage of urban female deaths to be truly due to over- reporting and not a mere data quality issue. We made further assessments of the data by computing age-standardized crude death rates (ASCDRs) and age-specific mortality rates (ASMR) for females in rural and urban areas. The urban female mortality was marginally higher; urban ASCDR was 12.65 compared with 12.38 for rural. The age-standardized ASMR for women over the age of 25 was much higher among urban females compared to rural females in the same age group (results not shown). Higher mortality in urban areas compared to rural areas is a bit odd given relatively better living conditions and access to health services in urban areas. The results could therefore reinforce the findings that urban female deaths were actually overstated.

Reporting of deaths is not only problematic if deaths are misreported overall, but also if reporting varies by age. This is even more important in the application of evaluations methods like the GGB and SEG [48]. A sensitivity analysis by Hill and colleagues found the methods to be more robust to age misreporting than variations in deaths coverage [25]. Deaths misreporting by age seemed to have been more pronounced in urban areas as shown by the curved shape of both the SEG and GGB-SEG instead of the expected horizontal line associated with constant deaths coverage by age.

Deaths were not the only data evaluated and in need of adjustment. Live births too needed significant upward adjustments for both rural and urban areas. Although the cohort PF ratio method is unlikely to be affected by changing fertility over time, it can be affected by changing mortality patterns and levels among reproductive-age women during the intercensal period [20, 49]. The assumption that there are no differences in fertility of women interviewed and those that died during the intercensal period maybe hard to sustain given the high mortality among reproductive age women in Zambia. Women who are highly fecund may also face greater risk of dying from pregnancy-related causes since they are more often pregnant. Without adjusting for under-coverage of live births in the 2010 census, partial adjustment resulted in much higher estimates of the PRMRatio overall and for rural areas (see Table 4), since live births form the denominator in the estimation. However, after adjusting for live births also, full adjustment resulted in lower estimates overall, in both rural and urban areas. Census-based adjusted TFRs were marginally higher than the DHS estimates for the period 3 years prior to the 2013/14 survey; 6.9 compared to 6.6 for rural areas, and 4.4 compared to 3.7 for urban areas respectively.

A major limitation of our study stems from the inability to adjust for rural-urban migration due to lack of data. Census-based migration data are affected by under-recording of actual migration volumes and directions during a lengthy intercensal period like 10 years [16].

Although the 2010 census indicated a positive rural-urban net migration of females aged 10–19 during the 2000–2010 intercensal period, the indicated volume was very small [50]. We therefore assumed zero migration effect in our application of the death distribution methods as required. However, the likelihood that this assumption was sustained for the 10-year intercensal period is low, and therefore violation could to some extent have affected the operations of the methods. This could have affected estimates for both rural and urban areas, as one area could have experienced net losses and the other net gains. However, this effect is not evident in the data evaluation results.

## Conclusion

Census-based estimates showed very high adult female mortality and particularly high pregnancy-related mortality in both rural and urban areas 12 months prior to the 2010 census. However, significant adjustments were necessary due to evidence of errors in the data on population age, age at death, live births, and deaths. The adjustments resulted in more plausible mortality differentials between rural and urban areas, albeit with still very high mortality in both. The adjusted PRMRatio was two times higher in rural areas than in urban areas. Future censuses should incorporate strategies for improving the quality of data collected.

## Abbreviations

ASAI: Age-sex accuracy index
ASCDR: Age standardized crude death rate
ASMR: Age-specific mortality rate
DHS: Demographic and Health Survey
GGB: General growth balance
MDG: Millennium Development Goal MMRatio Maternal mortality ratio
PMDF: Proportion of female deaths that are pregnancy-related
PRMRatio: Pregnancy-related mortality ratio
SEA: Standard enumeration area
SEG: Synthetic extinct generation
TFR: Total fertility rate
UN: United Nations
WHO: World Health Organization
_35_*q*_15_: Conditional probability of dying between exact age 15 and 50
_45_*q*_15_: Conditional probability of dying between exact age 15 and 60
_20_*q*_20_/_20_*q*_40_: Probability of dying between exact age 20 and 40 relative to dying between exact age 40 and 60
_30_*d*_10_/_20_*d*_40_: Number of life table deaths between exact age 10 and 40 relative to deaths between exact age 40 and 60
_20_*d*_20_/_20_*d*_40_: Number of life table deaths between exact age 20 and 40 relative to deaths between exact age 40 and 60
